# The Polysaccharide Capsule of *Streptococcus pneumonia* Partially Impedes MyD88-Mediated Immunity during Pneumonia in Mice

**DOI:** 10.1371/journal.pone.0118181

**Published:** 2015-02-20

**Authors:** Alex F. de Vos, Mark C. Dessing, Adriana J. J. Lammers, Alexander P. N. A. de Porto, Sandrine Florquin, Onno J. de Boer, Regina de Beer, Sanne Terpstra, Hester J. Bootsma, Peter W. Hermans, Cornelis van ‘t Veer, Tom van der Poll

**Affiliations:** 1 Center for Experimental and Molecular Medicine, Academic Medical Center, University of Amsterdam, Amsterdam, The Netherlands; 2 Center of Infection and Immunity Amsterdam (CINIMA), Academic Medical Center, University of Amsterdam, Amsterdam, The Netherlands; 3 Department of Pathology, Academic Medical Center, University of Amsterdam, Amsterdam, The Netherlands; 4 Radboud University Medical Center, Laboratory of Pediatric Infectious Diseases, Nijmegen, The Netherlands; Hannover School of Medicine, GERMANY

## Abstract

Toll-like receptors (TLR) and the downstream adaptor protein MyD88 are considered crucial for protective immunity during bacterial infections. *Streptococcus (S.) pneumoniae* is a human respiratory pathogen and a large majority of clinical pneumococcal isolates expresses an external polysaccharide capsule. We here sought to determine the role of pneumococcal capsule in MyD88-mediated antibacterial defense during *S. pneumonia* pneumonia. Wild type (WT) and *Myd88^-/-^* mice were inoculated intranasally with serotype 2 *S. pneumoniae* D39 or with an isogenic capsule locus deletion mutant (D39∆*cps*), and analysed for bacterial outgrowth and inflammatory responses in the lung. As compared to WT mice, *Myd88^-/-^* mice infected with D39 demonstrated a modestly impaired bacterial clearance accompanied by decreased inflammatory responses in the lung. Strikingly, while WT mice rapidly cleared D39∆*cps*, *Myd88^-/-^* mice showed 10^5^-fold higher bacterial burdens in their lungs and dissemination to blood 24 hours after infection. These data suggest that the pneumococcal capsule impairs recognition of TLR ligands expressed by *S. pneumoniae* and thereby partially impedes MyD88-mediated antibacterial defense.

## Introduction

*Streptococcus (S.) pneumoniae* frequently resides in the upper respiratory tract of humans and other related mammalian species [1]. At least 93 capsular serotypes of pneumococci exist in humans and once colonized by a new serotype, the pneumococcus can persist for weeks or even months without causing disease. In a subset of subjects, colonization leads to invasive infection, making *S. pneumoniae* the most common causative microorganism in community-acquired pneumonia (CAP) [2,3]. In the United States alone, the pneumococcus is responsible for more than half a million CAP cases each year, with a fatality rate of 5–7% [4]. Knowledge of host defense mechanisms that influence the outcome of pneumococcal pneumonia may aid in developing novel therapeutic strategies in an era of growing resistance of the pneumococcus against currently available antimicrobial agents [2,3].

Toll-like receptors (TLRs) play a central role in the initiation of cellular innate immune responses by virtue of their capacity to detect pathogens at either the cell surface or in lysosomes/endosomes [5,6]. Ten TLRs have been identified in humans and 12 in mice. Different TLRs recognize different conserved motifs expressed by pathogens called “pathogen associated molecular patterns”. TLRs can sense several of these patterns within *S. pneumoniae* and thereby contribute to the induction of an effective innate immune response. TLR2 has been implicated as the principal TLR in the recognition of *S. pneumoniae* [7–9], at least in part through an interaction with lipoteichoic acid, a constituent of the pneumococcal cell wall [10–12]. Nonetheless, TLR2 does not play a major role in containment of the infection during pneumococcal pneumonia [8,13]. TLR4 contributes to host defense during *S. pneumoniae* pneumonia through its capacity to recognize pneumolysin [14,15]. The interaction between pneumolysin and TLR4 has been found to limit the growth of pneumococci in the nasopharynx in a model of colonization [14,15] whereas TLR4 may also contribute to antibacterial defense during pneumococcal infection of the lower airways [16]. Finally, TLR9, which recognizes bacterial DNA, was found to be required to control bacterial growth and dissemination after induction of pneumococcal pneumonia [17]. All TLRs, except TLR3, rely on myeloid differentiation primary-response protein 88 (MyD88) for signaling. In accordance with a role for TLR signaling in host defense against pneumococcal pneumonia, MyD88 deficient (*Myd88* ^*-/-*^) mice were reported to show a profoundly enhanced growth of pneumococci and a strongly reduced survival after intranasal infection with serotype 4 or 19F *S. pneumoniae* strains [18].

At least 93 different serotypes of *S. pneumoniae* have been identified based on antigenic differences in their capsular polysaccharides. The capsule of *S.pneumoniae* is a major virulence factor, as illustrated by the fact that virtually all clinical isolates contain a capsule and that non-encapsulated pneumococcal strains are markedly less virulent in animals [19]. The pneumococcal capsule provides protection against phagocytosis [19], complement-mediated immunity [20] and opsonophagocytic killing [21]. Furthermore, the capsule markedly reduces capture of pneumococci by neutrophil extracellular traps [22]. Moreover, the capsule also prevents entrapment of *S. pneumoniae* in airway mucus and as a consequence limits mucus-mediated clearance [23]. The capsule potentially also interferes with innate immune responses by masking several TLR ligands associated with the pneumococcal cell wall, including lipoteichoic acid and lipopeptides [3,19]. Therefore, we here hypothesized that the pneumococcal capsule may impede TLR-mediated protective immunity during infection with *S. pneumoniae*. Hence, in the present study we investigated the host response during pneumonia caused by the serotype 2 strain D39 and an isogenic capsule locus (*cps*) deletion mutant of D39 (D39Δ*cps*) [24] in both wild type (WT) and *Myd88* ^*-/-*^ mice. Our findings reveal that the polysaccharide capsule partially protects pneumococci against MyD88-mediated immunity during pneumonia in mice.

## Materials and Methods

### Ethics statement

Experiments were carried out in accordance with the Dutch Experiment on Animals Act and approved by the Animal Care and Use Committee of the University of Amsterdam (Permit number DIX 100121).

### Mice

C57BL/6 WT mice were purchased from Harlan Sprague Dawley Inc. (Horst, The Netherlands) and *Myd88* ^-/-^ mice [25], backcrossed 7 times to a C57BL/6 genetic background, were generously provided by Dr. Shizuo Akira (Osaka University, Japan). All mice were bred and housed in specific pathogen-free rooms within the animal facility of the Academic Medical Center (Amsterdam, The Netherlands). Age (9–11 week old) and gender matched mice were used in all experiments.

### Bacteria

The *S. pneumoniae* strains used in this study were D39 and D39Δ*cps*, the isogenic capsule locus (*cps*) deletion mutant of WT D39 [24]. Both D39 and D39Δ*cps* were grown for 3–6 hours to mid-logarithmic phase at 37°C using Todd-Hewitt broth (Difco, Detroit, MI), supplemented with yeast extract (0.5%). Bacteria were harvested by centrifugation at 4000 rpm, and washed twice in sterile isotonic saline.

### Experimental design

Pneumonia was induced by intranasal administration of bacteria (total volume 50 μl) under light anaesthesia by inhalation of isoflurane (Abbott, Kent, UK) as described previously [8,12,26]. Mice were killed 6 or 48 hours after infection with D39 pneumococci (5 x 10^6^ colony forming units (CFU)), and 6 or 24 hours after infection with D39Δ*cps* bacteria (10^8^ CFU)(N = 6–8 per group at each time point). The infectious dose of D39Δ*cps* was chosen based on the fact that lower doses are rapidly cleared from the airways by normal WT mice [27]. Lung bacterial loads were determined as described earlier [8,12,26]. Briefly, mice were sacrificed and blood and lungs were collected. Lungs were homogenized at 4°C in 5 volumes of sterile isotonic saline with a tissue homogenizer (Biospect Products, Bartlesville, OK). Serial 10-fold dilutions in sterile isotonic saline were made from these homogenates (and blood), and 50 μl volumes were plated onto sheep-blood agar plates and incubated overnight at 37°C and 5% CO_2_.

### Histology

Lungs for histology were fixed in 10% formalin and embedded in paraffin. Four μm sections were stained with hematoxylin and eosin (HE) and analyzed by a pathologist who was blinded for groups. To score lung inflammation and damage, the entire lung surface was analyzed with respect to the following parameters: bronchitis, edema, interstitial inflammation, intra-alveolar inflammation, pleuritis and endothelialitis. Each parameter was graded on a scale of 0 to 4 with 0 as ‘absent’ and 4 as ‘severe’. The total “lung inflammation score” was expressed as the sum of the scores for each parameter, the maximum being 24. Sections were stained with FITC-labeled anti-mouse Ly6-C/G mAb (BD Biosciences, San Jose, CA) to determine granulocyte infiltration in the lung, as described before [27]. Ly6-C/G expression in the lung was quantified by digital image analysis and the amount of Ly6-C/G positivity was expressed as a percentage of the total surface area, as described [27].

### Assays

Lung homogenates were prepared as described [8,12,26]. Myeloperoxidase (MPO) was measured by ELISA (HyCult, Uden, The Netherlands). Tumor necrosis factor (TNF)-α, interleukin (IL)-1β, IL-6, macrophage inflammatory protein (MIP)-2 and cytokine-induced neutrophil chemoattractant (KC) were measured by ELISA (R & D Systems, Abingdon, UK).

### Statistical analysis

Statistical analysis was performed with GraphPad Prism version 4.00 for Windows (GraphPad Software, San Diego, CA). All data are given as means ± SEM. Differences between groups were analyzed using Mann-Whitney U test. P < 0.05 was considered to present a statistically significant difference.

## Results

### MyD88 especially limits bacterial growth and dissemination during pneumonia caused by non-encapsulated *S. pneumoniae*

**Fig 1:**
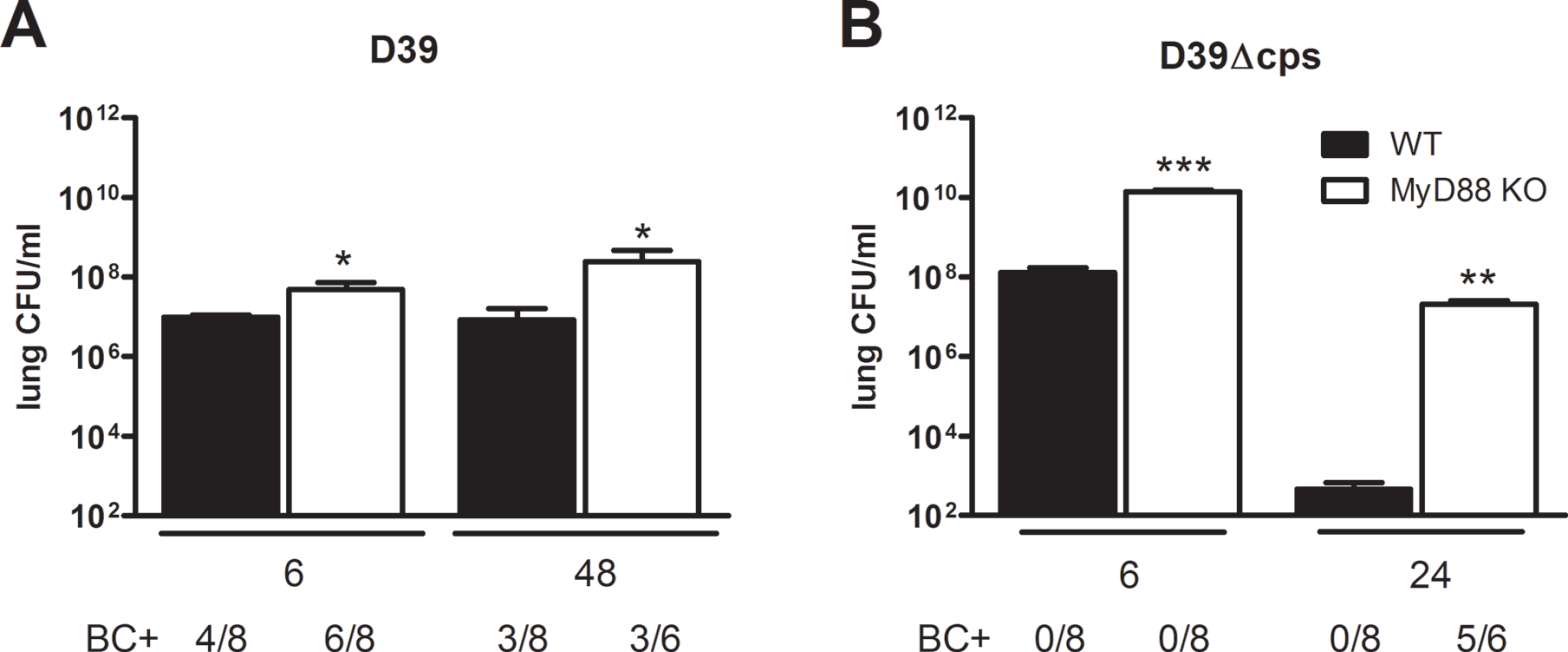
Enhanced susceptibility of Myd88^-/-^ mice for D39 or D39Δ*cps*. (A) Bacterial loads in lung homogenates of wild type mice (WT, black bars) and Myd88^-/-^ mice (MyD88 KO, white bars) at 6 and 48 hours after infection with 5x10^6^ CFU *S. pneumoniae* D39, and (B), 6 and 24 hours after infection with 1x10^8^ CFU D39Δ*cps*. BC+ indicates the number of positive blood cultures. Data are mean ± SEM. (N = 6–8 mice per group at each time point). *P<0.05, **P<0.005, ***P<0.0005 vs WT mice.

To determine the impact of the pneumococcal capsule on MyD88 mediated antibacterial defense, we infected WT and *Myd88* ^*-/-*^ mice with either WT encapsulated D39 *S. pneumoniae* or with isogenic non-encapsulated D39Δ*cps* pneumococci via the airways. After instillation of D39 we euthanized mice 6 or 48 hours after infection for measurements of bacterial loads in lungs and blood, seeking to obtain insight into the role of MyD88 in the early and late host response (Fig. 1A). At both time points, *Myd88* ^*-/-*^ mice had higher bacterial loads in their lungs than WT mice (P<0.05), although the differences between mouse strains were relatively modest. MyD88 deficiency was not associated with enhanced dissemination of D39 to the circulation (Fig. 1A). Since we previously found that WT mice cleared high D39Δ*cps* doses within 24 hours [27], *Myd88* ^*-/-*^ and WT mice were sacrificed 6 or 24 hours after infection with D39Δ*cps*, again seeking to obtain information about the early and late host response (Fig. 1B). Analysis of bacterial outgrowth in the lung showed that *Myd88* ^*-/-*^ mice had a strongly diminished capacity to clear D39Δ*cps*: whereas WT mice demonstrated a >10^5^-fold reduction in pulmonary bacterial loads between 6 and 24 hours after infection, the reduction in *Myd88* ^*-/-*^ mice was only ~10^2^-fold in this time frame. At both time points the lungs of MyD88^-/-^ mice contained much higher burdens of D39Δ*cps* as compared to the lungs of WT mice (P<0.005); at 24 hours after infection the average difference was as high as 10^5^-fold. Importantly, dissemination of D39Δ*cps* to the circulation was only found in *Myd88* ^*-/-*^ mice, not in WT mice, 24 hours after infection (P<0.005). These results indicate that MyD88 has a stronger impact on antibacterial defense after infection with the non-encapsulated D39Δ*cps* strain.

### MyD88 mediates the early inflammatory response in the lung during pneumonia caused by capsulated and non-encapsulated *S. pneumoniae*

**Fig 2:**
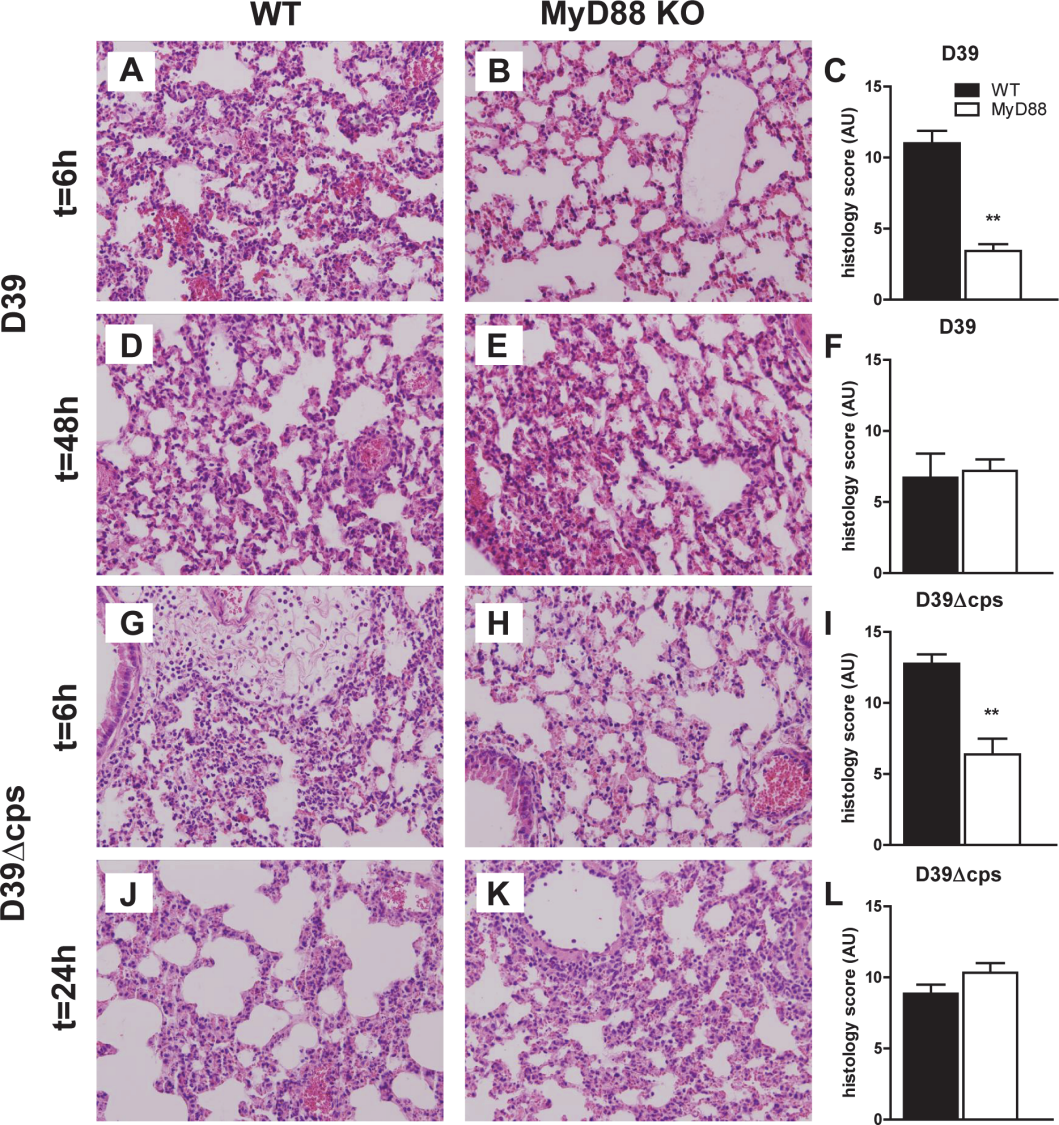
Lung pathology in WT and Myd88^-/-^ mice during infection with capsulated and unencapsulated D39. Representative lung sections obtained 6 or 48 h after induction of pneumococcal pneumonia with D39 in WT (A and D) and Myd88^-/-^ mice (B and E) and 6 or 24 h after inoculation with D39Δ*cps* in WT mice (G and J) and MyD88^-/-^ mice (H and K). Haematoxylin and eosin stainings show different levels of inflammation. Original magnification 20x. Findings were quantified by histology scoring, as described in methods section for wild type (WT, black bars) and Myd88^-/-^ mice (MyD88 KO, white bars). Data are mean ± SEM (N = 5–8 mice per group at each time point). **P<0.005 vs WT mice.

The induction of a brisk inflammatory response in the lungs is of great importance for containment of the infection during pneumococcal pneumonia [3,28]. To obtain insight into the mechanism underlying the role of MyD88 in limiting bacterial growth after infection with D39 and D39Δ*cps* pneumococci, we prepared lung tissue slides from mice at different time points after infection with either bacterial strain and analysed specific histological features characteristic for bacterial pneumonia. Lung pathology developed quickly in WT mice after infection with D39 (Fig. 2A and C), and was characterised by pleuritis, interstitial inflammation, endothelialitis, bronchitis and edema. In contrast, *Myd88* ^*-/-*^ mice demonstrated strongly reduced lung inflammation early after infection with D39 (P<0.01 vs WT mice), caused by reduced pleuritis, interstitial inflammation, bronchitis, endothelialtis and edema (Fig. 2B-C). At 48 hours after inoculation, the extent of lung pathology had decreased in WT mice and increased in *Myd88* ^*-/-*^ mice; the difference between mouse strains was not observed anymore (Fig. 2D-F). In spite of the strongly reduced virulence of D39Δ*cps*, this pneumococcal strain elicited a marked inflammatory response in the lungs of WT mice. The extent of lung inflammation was much lower in *Myd88* ^*-/-*^ mice at 6 hours (P<0.005 versus WT mice), caused by reduced interstitial inflammation, endothelilitis, bronchitis and edema (Fig. 2G-I). At 24 hours after infection with D39Δ*cps*, there was no difference in lung pathology between WT and *Myd88* ^*-/-*^ mice (Fig. 2J-L).

**Fig 3:**
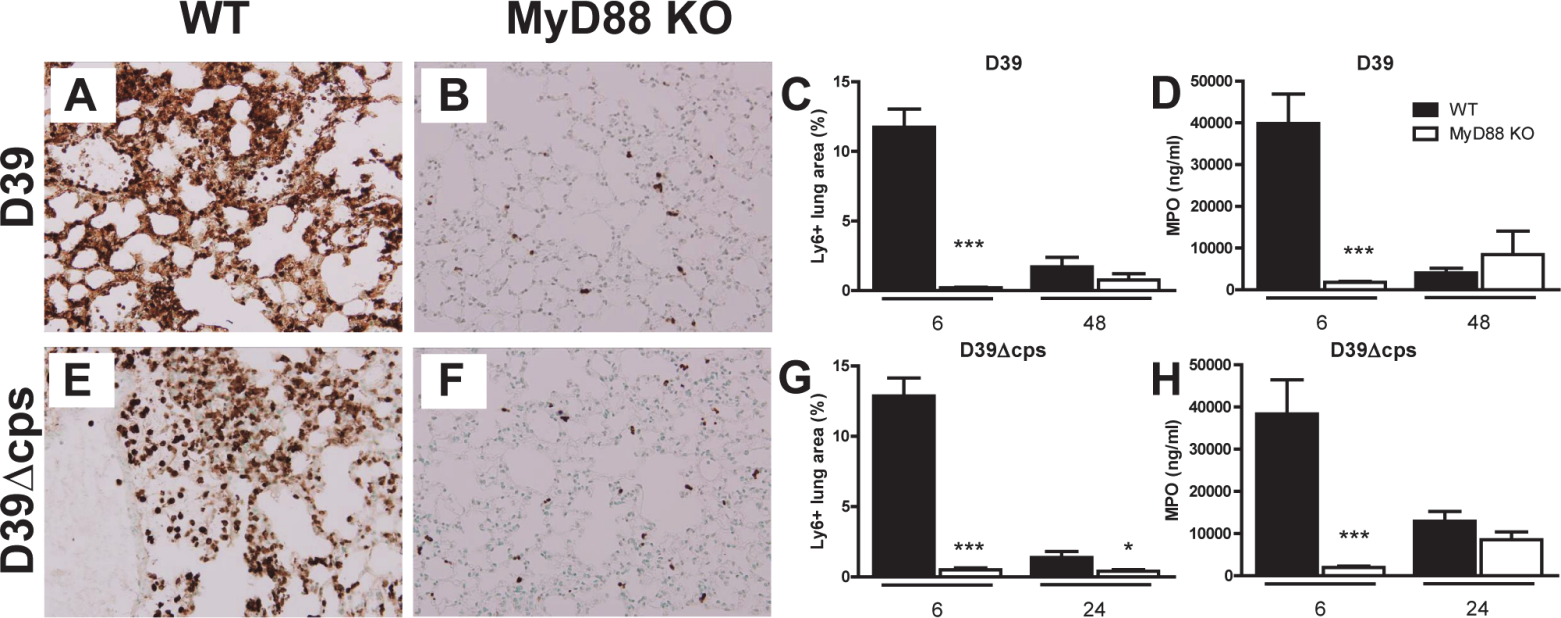
Reduced neutrophil infiltration in the lung in Myd88 ^-/-^ mice during infection with D39 or D39Δ*cps*. Representative Ly6-C/G stainings of lung sections obtained 6 h after induction of pneumococcal pneumonia with D39 in WT and Myd88^-/-^ mice (A and B) and with D39Δ*cps* in WT mice and Myd88^-/-^ mice (E and F). Original magnification 20x. Percentage lung area with Ly6-C/G-positive neutrophils was quantified by digital image analysis as described in methods section for wild type (WT, black bars) and Myd88^-/-^ mice (MyD88 KO, white bars)(C and G). Levels of MPO in lung homogenates of wild type (WT, black bars) and Myd88^-/-^ mice (MyD88 KO, white bars) inoculated with D39 at 6 and 48 hours after challenge, or inoculated with D39Δcps (D and H) at 6 and 24 hours after challenge were determined by ELISA. Data are mean ± SEM (N = 5–8 mice per group at each time point). *P<0.05, ***P<0.0005 vs WT mice.

In accordance with these histological data, D39 triggered an early and transient neutrophil influx into the lungs of WT mice, as reflected by a marked increase in the number of Ly6+ cells in tissue slides and elevated concentrations of MPO in whole lung homogenates at 6 hours post infection, which was strongly reduced in *Myd88* ^*-/-*^ mice (Fig. 3A-D, both P<0.0005). At 48 hours after infection with D39, neutrophil counts in lungs had declined strongly in WT mice and were not different from those in *Myd88* ^*-/-*^ mice. Similarly, Ly6-C/G staining and MPO measurements revealed that D39Δ*cps* induced a rapid and transient influx of neutrophils into lung tissue of WT mice at 6 after inoculation, which was not observed in *Myd88* ^*-/-*^ mice (Fig. 3E-H, both P<0.0005). At 24 hours post D39Δ*cps* inoculation, neutrophil infiltration in the lung had strongly decreased in WT mice; at this time point *Myd88* ^*-/-*^ mice still had fewer Ly6+ cells in lung tissue when compared with WT mice (P<0.05).

### Role of MyD88 in the cytokine and chemokine response in the lung during pneumonia caused by capsulated and nonencapsulated *S. pneumoniae*

**Fig 4:**
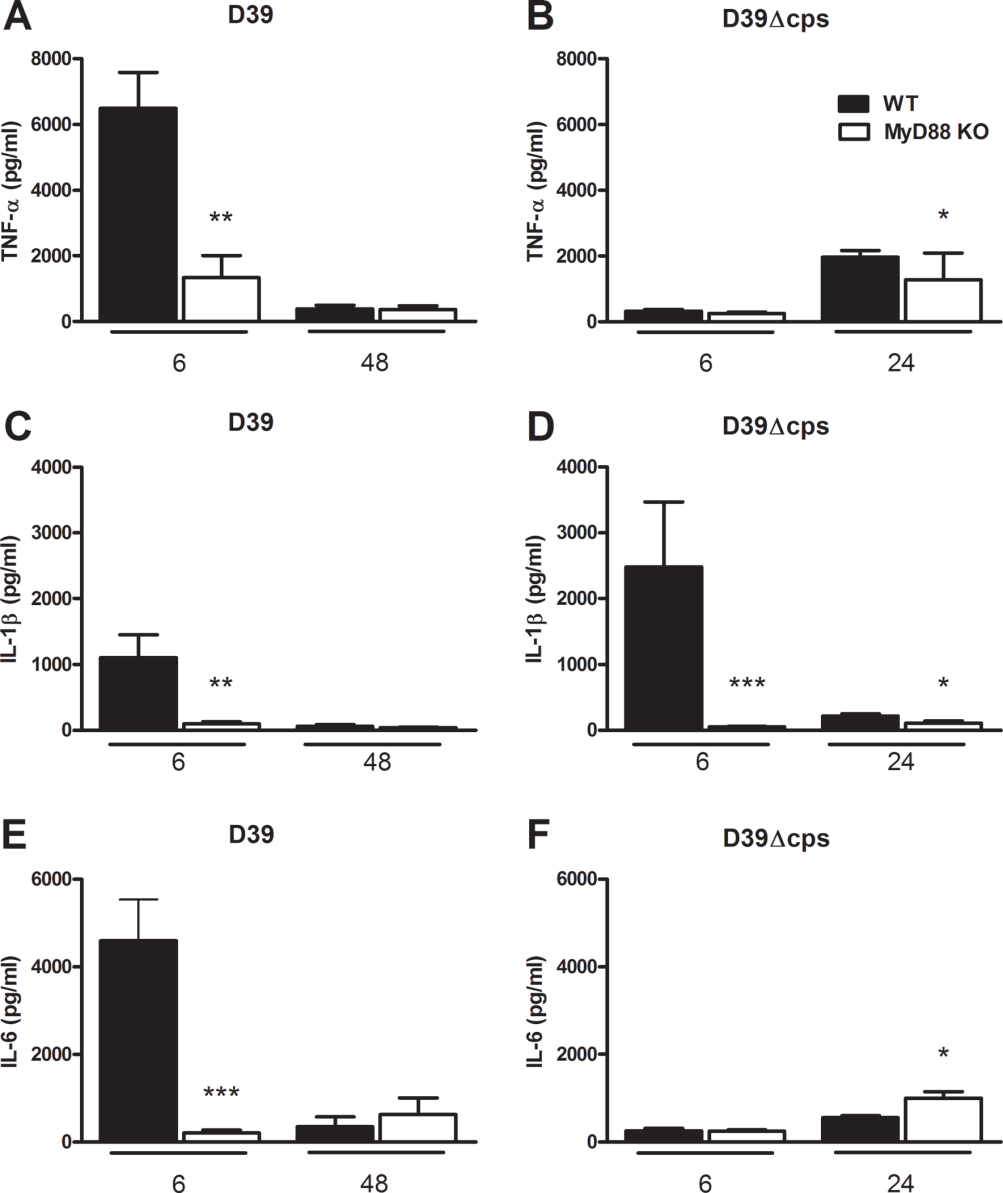
Reduced cytokine production in the lung of Myd88^-/-^ mice during infection with D39 or D39Δ*cps*. Lung levels of TNF-α (A and B), IL-1β (C and D) and IL-6 (E and F) of wild type (WT, black bars) and Myd88^-/-^ mice (MyD88 KO, white bars) at 6 and 48 hours after inoculation with D39 (A/C/E) or at 6 or 24 hours post inoculation of D39Δ*cps* (B/D/F). Data are mean ± SEM (N = 6–8 mice per group at each time point). *P<0.05, **P<0.005, ***P<0.0005 vs WT mice.

**Fig 5:**
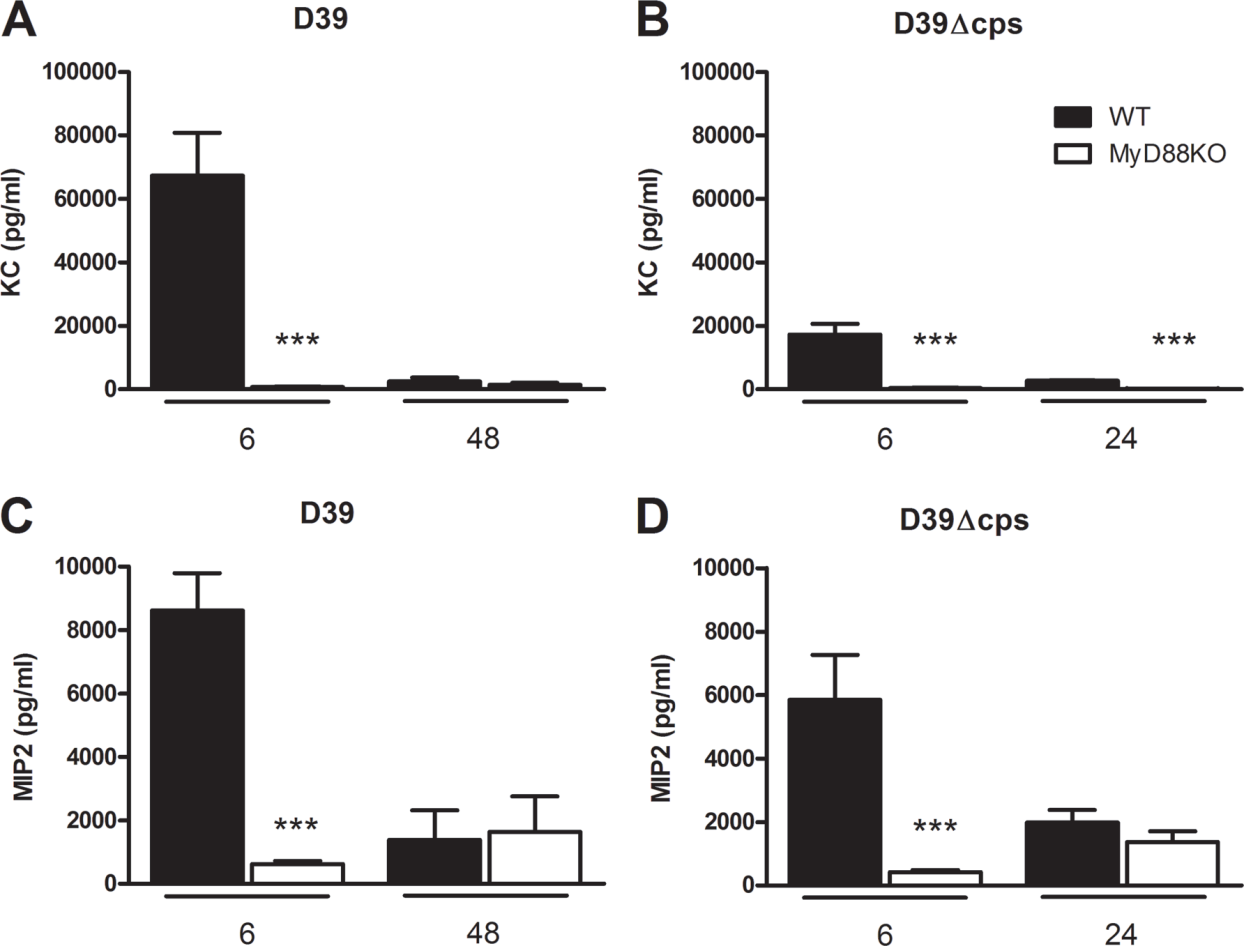
Reduced chemokine production in the lung of Myd88^-/-^ mice during infection with D39 or D39Δ*cps*. KC/CXCL1 (A and B) and MIP2/CCL2 (C and D) of wild type (WT, black bars) and Myd88^-/-^ mice (MyD88 KO, white bars) at the indicated time points after inoculation with D39 (A/C) or at 6 or 24 hours post inoculation D39Δ*cps* (B/D). Data are mean ± SEM (N = 6–8 mice per group at each time point). ***P<0.0005 vs WT mice.

Local production of cytokines and chemokines plays an eminent role in the orchestration of the inflammatory response in the lungs during pneumococcal pneumonia [3]. We therefore considered it of interest to examine the impact of MyD88 deficiency on the concentrations of proinflammatory cytokines (TNF-α, IL-1β, IL-6) and CXC chemokines (KC, MIP-2) after infection with D39 or D39Δ*cps* (Figs. 4 and 5). Inoculation with D39 induced a brisk cytokine and chemokine response at 6 hours with much lower levels at 48 hours. Importantly, relative to WT mice, *Myd88* ^*-/-*^ mice showed a markedly attenuated cytokine and chemokine response at 6 hours after infection with D39 (P<0.005 in all cases; Figs. 4 and 5). Strikingly, the capacity of D39Δ*cps* to induce the production of most cytokines in the lung was rather low as compared to D39 (Fig. 4), with pulmonary TNF-α and IL-6 levels at 6 hours after inoculation of D39Δ*cps* in WT mice significantly reduced as compared to D39 (P<0.0005 in both cases). In contrast, IL-1β concentrations were strongly elevated in WT mice, but not in *Myd88* ^*-/-*^ mice 6 hours after inoculation of D39Δ*cps* (P<0.0005; Fig. 4D). Besides IL-1β, D39Δ*cps* also induced an early and transient increase of MIP-2 levels in the lung of WT mice, but not in *Myd88* ^*-/-*^ mice (Fig. 5D). The induction of KC in the lung of WT mice by D39Δ*cps* was markedly reduced as compared to D39 (P<0.05; Fig. 5).

## Discussion

The polysaccharide capsule is a major virulence factor of *S.pneumoniae* rendering bacteria resistant to the lethal effects of complement, shielding bacteria from opsonophagocytosis and killing, inhibiting capture by neutrophil extracellular traps and limiting mucus-mediated clearance [19–23]. Studies with capsular switch mutants revealed that the capsule rather than the genetic background influences resistance of *S. pneumoniae* to host immune responses and virulence in mice [20,21,29]. Since TLR ligands are masked by capsular polysaccharides in certain bacteria [30,31], we here studied the role of the pneumococcal capsule in MyD88-mediated antibacterial defense against *S. pneumoniae* using the serotype 2 strain D39 and D39Δ*cps*. While the present finding of enhanced bacterial growth of D39 in *Myd88* ^*-/-*^ mice is in accordance with earlier results after infection with serotype 4 or 19F *S. pneumoniae* [18], we demonstrate that MyD88 plays a more important role in limiting pneumococcal growth after infection with D39Δ*cps*. Whereas *Myd88* ^-/-^ mice infected with D39 displayed a modestly impaired bacterial clearance, these knock-out mice showed 10^5^-fold higher bacterial burdens in their lungs 24 hours after infection with D39Δ*cps* as compared to WT mice. These data suggest that part of the virulence of encapsulated pneumococci relies on the capacity of the capsule to impair recognition of TLR ligands expressed by *S. pneumoniae*.

In line with previous studies [27,32,33], loss of the capsule greatly attenuated the virulence of D39 in WT mice. Analysis of the cytokine and chemokine responses in the lung showed that D39, but not D39Δ*cps* pneumococci despite higher bacterial numbers, triggered the production of TNF-α, IL-6 and KC in the lung 6 hours after infection. These findings indicate that the capsule is a major factor in the early induction of cytokines at the primary site of infection with *S.pneumoniae*. Although pneumococcal polysaccharides do not directly trigger TLR signaling, the early cytokine response evoked by D39 was strongly attenuated in *Myd88* ^-/-^ mice. These results suggest that pneumococcal polysaccharides or components associated with polysaccharides on the outmost surface of the bacterium enhance TLR dependent cytokine release elicited by pneumococcal TLR ligands such as lipoteichoic acid, pneumolysin and DNA. A possible mechanism may be that capsular polysaccharides facilitate binding of pneumococci to cells (e.g. via C-type lectin receptors) and thereby augment cytokine production. Further studies are required to determine the role of the pneumococcal capsule in induction of distinct inflammatory mediators in the lungs during infection with D39. In contrast to D39, D39Δ*cps* did not induce TNF-α and IL-6, and triggered low levels of KC in the lung 6 hours after intranasal infection. D39Δ*cps* inoculation, however, induced significant expression of IL-1β and MIP-2 in a MyD88-dependent manner. The mechanism underlying the induction of cytokine synthesis by D39Δ*cps* is unclear so far. Production of IL-1β is induced by TLR, and subsequent activation of intracellular PRRs leads to the formation of the inflammasome required to cleave the immature pro-form into the mature secreted form of this cytokine [34]. Activation of cytosolic PRRs occurs after invasion or after escape of microbes or microbial products from phagosomes, although the exact processes leading to the activation of intracellular PRRs are still not completely disentangled [34]. Unencapsulated pneumococci, unlike capsulated pneumococci, are readily phagocytosed by macrophages [35,36], and phagocytosis of unencapsulated serotype 4 pneumococci by dendritic cells was recently found to result in a significant secretion of IL-1β [37]. Despite low levels of TNF-α, IL-6 and KC in the lung early after infection, D39Δ*cps* induced profound lung inflammation like D39, as determined by semi-quantitative pathology scores of lung tissue slides and by measuring neutrophil influx. These data suggest that IL-1β and MIP-2 are among the driving mediators of the inflammatory response evoked by this pneumococcal strain.

The serotype of the infecting *S. pneumoniae* is an important risk factor for the occurrence of invasive disease and mortality [38]. As such, it is imperative to examine host defense mechanisms during infection with different pneumococcal serotypes. Previously, we and others have shown that recognition of *S. pneumoniae* by TLR2, TLR4 and TLR9 contributes to the pulmonary host defense response against serotype 2, serotype 3 and serotype 4 pneumococci [8,12,16,17]. The findings of the present study with a serotype 2 pneumococcal strain and an unencapsulated mutant of this strain confirm the previously reported critical role of MyD88 in innate immunity against serotype 4 and 19F pneumococci [18]. Importantly, however, the more prominent role of MyD88 in host defense against unencapsulated versus encapsulated serotype 2 *S. pneumoniae*, as documented in the current study, suggests that the pneumococcal capsule impairs recognition of TLR ligands expressed by this pathogen. This mechanism may contribute to the well-established role of the capsule in the virulence of *S. pneumoniae*.
